# Innovative 4D FreeBreathing technique in pediatric abdominal MRI improves feasibility and image quality

**DOI:** 10.1007/s00330-025-11577-2

**Published:** 2025-04-16

**Authors:** Patricia Tischendorf, Laura Beck, Tobias Krähling, Jan H. Lange, Walter Heindel

**Affiliations:** 1 Clinic for Radiology, University of Münster and University Hospital Muenster, Münster, Germany; 2 Department of Anesthesiology, University of Münster and University Hospital Münster, Münster, Germany

**Keywords:** Magnetic resonance imaging, Infant, Children, Abdomen, Anesthesia

## Abstract

**Objectives:**

To compare the feasibility and imaging quality of a golden angle radial stack-of-stars dynamic three-dimensional free-breathing T1w turbo field echo acquisition (4D FreeBreathing) with a conventional dynamic cartesian breath-hold T1w sequence in young children undergoing abdominal magnetic resonance imaging (MRI).

**Materials and methods:**

Fifty consecutive pediatric patients (34 females; 3.4 ± 2.0 years) underwent abdominal MRI: 25 were examined with 4D FreeBreathing and 25 with conventional dynamic T1w sequence. The image quality was evaluated subjectively on a 5-point scale by two radiologists. Interobserver agreement, as well as signal-to-noise ratio for arterial (SNRart) and portal venous (SNRpv) phases, were evaluated separately. Additionally, the image quality of 4D FreeBreathing sequence was compared to a non-dynamic post-contrast radial stack-of-stars free-breathing T1w fast field echo acquisition (3D T1w Vane mDixon). Interobserver agreement of both assessors was calculated using quadratic weighted Cohen’s kappa test (ϰ), while independent samples Student’s *t*-test was employed to compare mean SNR values among the two groups.

**Results:**

Using 4D FreeBreathing, SNRart and SNRpv were significantly higher from 500 ± 170 and 550 ± 160 to 900 ± 210 and 820 ± 260 (*p* < 0.001); the diagnostic image quality increased from 77.6 to 89.6%; respiratory artifacts decreased from 22.4 to 10.4%, with an almost perfect interobserver agreement. Compared to 3D T1w Vane mDixon sequence, SNR and image quality were equal.

**Conclusion:**

4D FreeBreathing pediatric abdominal MRI improves the feasibility and image quality compared to conventional dynamic exams while showing an image quality equivalent to post-contrast 3D T1w Vane mDixon.

**Key Points:**

***Question*** *During dynamic abdominal MRI in young children, it is important to conduct a brief yet robust examination without respiratory artifacts*.

***Findings*** *4D FreeBreathing MRI technique for pediatric abdominal imaging enhances both image quality and feasibility when compared to conventional dynamic scans that require breath-holding*.

***Clinical relevance*** *Dynamic abdominal MRI using the 4D FreeBreathing sequence provides significant benefits for pediatric patients. The absence of breath-holding requirements improves patient cooperation, reduces the need for general anesthesia, and results in higher-quality diagnostic images*.

**Graphical Abstract:**

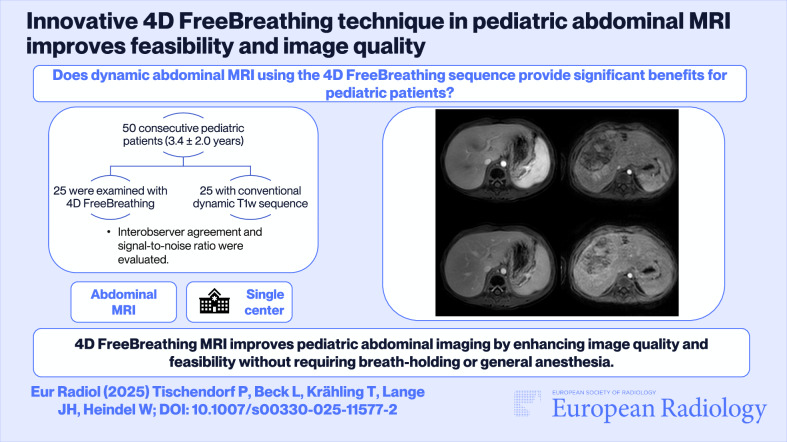

## Introduction

Magnetic resonance imaging (MRI) plays a crucial role in abdominal diagnostics for pediatric patients [1]. This is primarily due to the absence of ionizing radiation and the excellent soft tissue contrast enabling an objective representation of organs compared to ultrasound; it is especially necessary in oncologic studies [2–4]. Performing an MRI examination of young children, however, may be challenging, mainly due to motion artifacts, respiratory artifacts, the need for general anesthesia and the extended examination time [5, 6]. Therefore, it remains essential to continually seek solutions to improve these aspects, ensuring the best possible examination for young patients. MR imaging in pediatric patients is often performed with general anesthesia to decrease motion and respiratory artifacts [7], which results in additional risks for young pediatric patients [8]. Previous studies have already demonstrated the superiority of radial imaging over cartesian imaging in infants [9], as well as confirming the advantages of contrast-enhanced radial free-breathing T1w sequences [10, 11]. In routine clinical practice, dynamic contrast-enhanced T1w acquisition is needed, among other things, in young children with liver-originating neoplasia, like hepatoblastoma [12, 13]. However, other abdominal tumors, such as neuroblastoma and nephroblastoma, may benefit from dynamic abdominal MRI, especially for tumor differentiation or the representation of less perfused tissues or necrotic areas [14, 15]. Even pediatric patients without sedation or general anesthesia who understand and follow oral commands may have difficulty suspending respiration up to 20 s, resulting in suboptimal or non-diagnostic images [16, 17]. However, respiratory-navigated sequences can result in unpredictable acquisition times that may limit the diagnostic utility of contrast-enhanced abdominal imaging and also decrease imaging time. Therefore, a radial 3D T1w sequence that uses the stack-of-stars scheme for acquisitions is a good choice. Recent studies have shown that contrast-enhanced free-breathing radial sequence results in a superior image quality compared to the conventional cartesian breath-hold in pediatric patients [18].

However, there is so far less data on dynamic free-breathing radial stack-of-stars T1w sequences in young pediatric patients. Therefore, the purpose of our study was to compare the feasibility and image quality of the innovative golden angle radial stack-of-stars dynamic three-dimensional free-breathing T1w turbo field echo (4D FreeBreathing) sequence in young pediatric patients to a conventional dynamic contrast-enhanced protocol with breath-hold. In order to confirm the high image quality and to potentially save on examination time, the new 4D FreeBreathing sequence was additionally compared with a non-dynamic post-contrast radial stack-of-stars free-breathing T1w fast field echo sequence (3D T1w Vane mDixon), in order to consider whether the latter can be omitted.

## Materials and methods

### Study population

This study was approved by the local ethics committee. We conducted a retrospective review of dynamic MRI of the abdomen in a consecutive cohort of 50 young pediatric patients. These examinations were carried out over a two-year period spanning the period from December 2020 to December 2022, using a dynamic cartesian breath-hold T1w sequence and an additional one-year period from December 2022 to December 2023 using the newly available 4D FreeBreathing sequence. The pediatric patients were categorized into two groups, depending on whether the 4D FreeBreathing sequence was used (4DFB) or not (BH). In addition, the need for sedation or general anesthesia was retrieved from clinical records and documented.

### MR examinations

MRI was performed up to July 2022 on a 1.5-T Achieva Nova Dual (Philips Healthcare) and since September 2022 on a 1.5-T Achieva SmartPath to dStream (Philips Healthcare). All sequence parameters such as slice thickness, repetition time, echo time, flip angle, slice distance, field-of-view and recon pixel size were recorded in the DICOM (Digital Imaging and Communications in Medicine) metadata dictionary for every sequence, and they are shown in Table 1. Standard imaging protocol of group 4DFB included axial T2w MultiVane, coronal T2w spectral presaturation with inversion recovery (SPIR), axial diffusion-weighted imaging (DWI), axial dynamic 4D FreeBreathing with 4 phases (native, arterial, portal venous and equilibrium) and axial post-contrast 3D T1w Vane mDixon, while the standard image protocol of group BH included axial T2w MultiVane, coronal T2w SPIR, axial DWI, cartesian axial dynamic 3D T1w mDixon and axial post-contrast T1w mDixon. The DICOM data files for each MRI examination were reviewed, and the start time of the initial localizer sequence, the end time of the final sequence, as well as the start and end times of the dynamic T1w contrast sequences, were documented.

**Table 1:** Magnetic resonance imaging (MRI) sequence parameters

MRI parameters	4D FreeBreathing	Cartesian dynamic T1w mDixon	3D T1w Vane mDixon
Recon pixel size (mm)	0.74 × 0.74	0.94 × 0.94	0.78 × 0.78
Field of view (cm)	250	300	250
Repetition time/echo time (ms)	4.2/−1.8	5.9/−1.8 + 4.0	4.8/−2.4 + 4.7
Flip angle (°)	15	15	10
Number of averages	1	1	1
Slice thickness (mm)	5	4	4
Spacing between slices (mm)	2.5	2	2
Respiratory motion compensation technique	Built-in respiratory soft gating	Breath-hold (10 s)	Diaphragm navigated
Median scan time (s)	220	200	150

### Image quality data

The image quality was assessed both subjectively and objectively by two independent radiologists with 10 years’ (P.T.) and 12 years’ (L.B.) experience. These assessors were blinded to patient particulars and the scanning approach. Each reader independently recorded observations with respect to the following image quality parameters on a 5-point Likert scale: overall image quality, hepatic edge sharpness, hepatic vessel clarity and respiratory motion robustness. For each parameter, 1 represented the lowest quality score—unacceptable overall image quality, undetectable parenchymal edge sharpness, unreadable parenchymal vessel clarity and respiratory artifact degradation—while 5 represented the highest image quality—excellent overall quality, absence of edge and vessel blur, and no respiratory artifact in accordance with criteria outlined by Ichikawa et al [19]. Additionally, the presence or absence of respiratory artifacts in the examination sequences was documented, along with any pathological findings detected. The signal-to-noise ratio (SNR) was quantified for dynamic T1w sequences in both groups and also for 3D T1w Vane mDixon (group 4DFB) of MRI examination involving the placement of a region of interest over a constant point in the liver, and determining the mean signal intensity. The SNR was calculated using the formula: SNR = mean signal of tissue / standard deviation of background noise. The SNR of the arterial (SNRart) and portal venous (SNRpv) phases were scored and evaluated separately.

### Statistical methods

Data were collected using Microsoft Excel (Microsoft Corporation), and standard descriptive statistics were computed. The subjective image quality was evaluated by calculating the interobserver agreement of both assessors using quadratic weighted Cohen’s kappa test (ϰ). Values of ϰ were interpreted in the following way: ϰ < 0.20, slight agreement; ϰ = 0.21–0.40, fair agreement; ϰ = 0.41–0.60, moderate agreement; ϰ = 0.61–0.80, substantial agreement; ϰ = 0.81–1.0, almost perfect agreement. The Kolmogorov-Smirnov test was applied to assess the normality of data distribution. Normally distributed data were analyzed by means of the Student’s *t*-test. In any case of unequal variances, the Wilcoxon signed-rank test was used. A *p*-value < 0.05 was considered statistically significant. The independent samples Student’s *t*-test was employed to compare mean SNR values among the two groups. All statistical analyses were conducted using IBM SPSS Statistics Version 29.0 (IBM Corporation). The data were anonymized and treated appropriately with respect to local data protection guidelines.

### Anesthetic protocols

Airway management decisions were made by the attending anesthesiologists, based on patient-specific factors and also the fact that 4D FreeBreathing examination does not require breath-holding. When breath-holding was required in children under general anesthesia, the anesthesiologists induced apnea. Our anesthesiologists perform sedation with dexmedetomidine and propofol to allow the children to breathe spontaneously. In the case of general anesthesia, sufentanil and propofol are given for induction, and general anesthesia is maintained with sevoflurane.

## Results

### Patients’ characteristics

The study population comprised 50 pediatric patients (34 females, 16 males; 3.4 years ± 2.0 years). The most common reason for imaging was neuroblastoma (16/50), followed by nephroblastoma (12/50) and hepatoblastoma (8/50). 22 pediatric patients were examined at the initial presentation and 28 for response assessment. There was no significant difference between both groups (Table 2).

**Table 2:** Patients’ characteristics and imaging time of both groups

	All	Group 4DFB	Group BH	*p*-value
Age (years)	3.4 ± 2.0	2.9 ± 1.7	3.8 ± 2.2	0.97
Female	16	18	17	0.83
Nephroblastoma	12	6	6	1
Neuroblastoma	16	9	7	0.16
Hepatoblastoma	8	3	5	0.16
Other indication	14	7	7	1
Scanned at initial presentation	22	10	12	0.81
Scanned for response assessment	28	14	14	1
Sedation	18	16	2	< 0.001
General anesthesia	25	7	18	< 0.001
No anesthesia	7	2	5	0.11
Dynamic T1w imaging time (min)	3.3 ± 0.7	3.4 ± 0.5	3.2 ± 1.2	0.21
Examination time (min)	39 ± 11	35 ± 9	42 ± 12	0.03
Examination time (min) general anesthesia	40 ± 11	35 ± 8	41 ± 12	0.03
Examination time (min) sedation	37 ± 10	34 ± 9	50 ± 24	< 0.001
Examination time (min) no anesthesia or sedation	40 ± 11	38 ± 6	40 ± 12	0.2

*4DFB* patient group using 4D FreeBreathing sequence, *BH* patient group using conventional dynamic T1w sequence with breath-hold

### Examination times and image quality

The mean dynamic T1w imaging times of all subjects were 3.4 ± 0.5 min in group 4DFB and 3.2 ± 1.2 min for group BH (*p* = 0.21), while total imaging time in group 4DFB was lower with 35 ± 9 min vs. 42 ± 12 min compared to the BH group (*p* = 0.03).

**Fig. 1:**
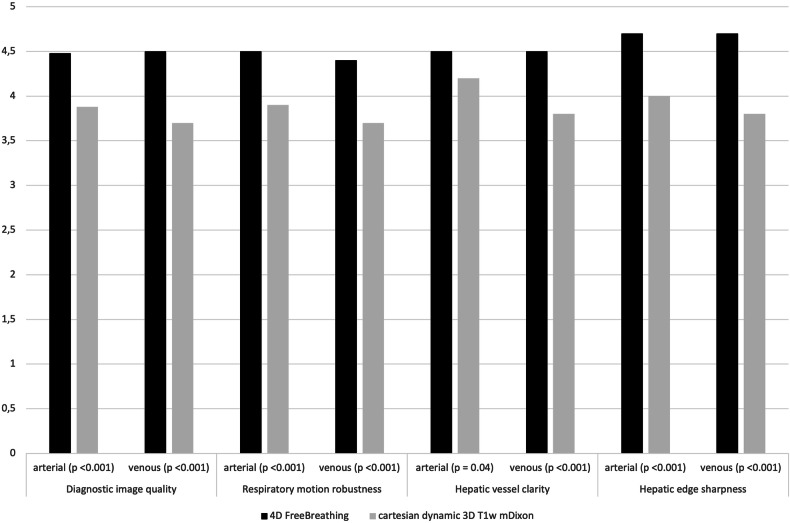
Average data of overall subjective diagnostic image quality for both readers 4D FreeBreathing versus cartesian dynamic 3D T1w mDixon

Using the 4D FreeBreathing sequence, SNRart and SNRpv were significantly higher at 900 ± 210 and 820 ± 260 versus 500 ± 170 and 550 ± 160 (*p* < 0.001) compared to the conventional dynamic exams with breath-hold. Subjective image quality of both the arterial and portal venous phase was rated in favor of the group examined with the 4D FreeBreathing. Figure 1 shows the average data of overall subjective image quality for both readers. Compared to the conventional breath-hold exam, the diagnostic image quality for the 4D FreeBreathing increased from 77.6 to 89.6%, whereas respiratory artifacts decreased from 22.4 to 10.4%.

**Fig. 2:**
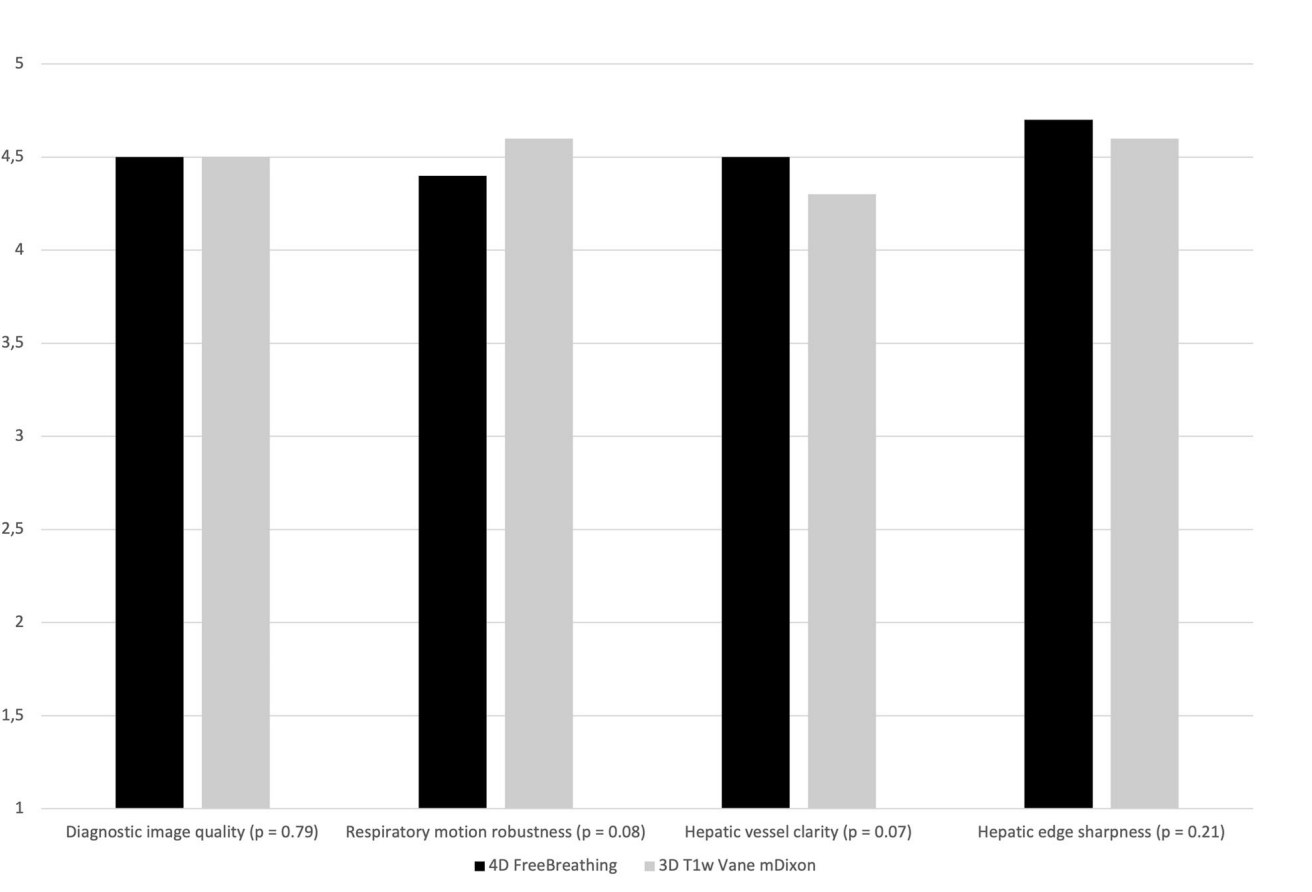
Average data of overall subjective diagnostic image quality for both readers 4D FreeBreathing versus 3D T1w Vane mDixon

**Fig. 3:**
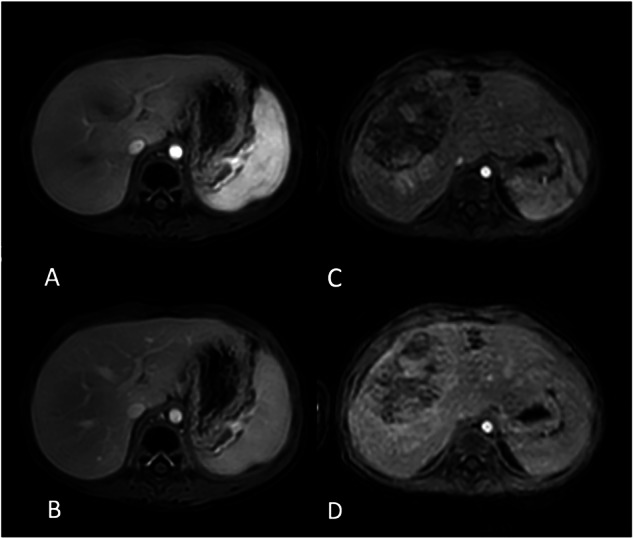
A 3-year-old girl with nephroblastoma using the 4D FreeBreathing in arterial phase (**A**) and portal venous phase (**B**). A 2-year-old girl with hepatoblastoma undergoing cartesian dynamic 3D T1w mDixon in arterial (**C**) and portal venous (**D**) phase. The diagnostic image quality was scored as follows by reader 1 (R1) and reader 2 (R2) using a 5-point Likert scale, with 5 being the best; **A** R1—4 and R2—5, **B** R1—4 and R2—5, **C** R1—3 and R2—3, **D** R1—3 and R2—3

**Fig. 4:**
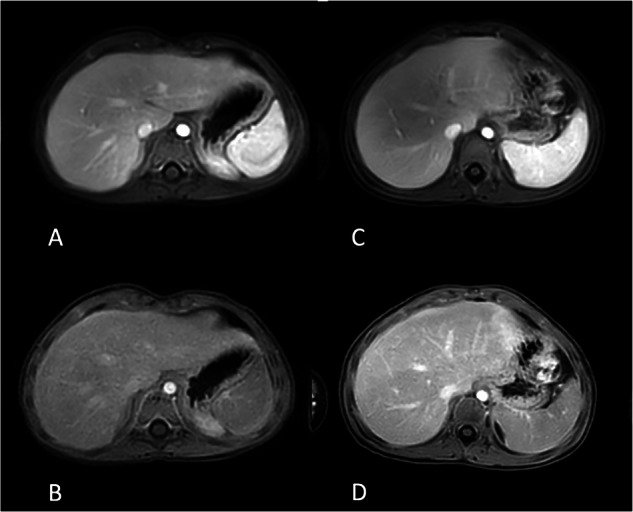
**A** A 3-year-old boy with nephroblastoma using 4D FreeBreathing in portal venous phase in comparison to 3D T1w Vane mDixon (**B**). A 3-year-old girl with nephroblastoma examined with 4D FreeBreathing in portal venous phase (**C**) and 3D T1w Vane mDixon (**D**). The diagnostic image quality was scored as follows by reader 1 (R1) and reader 2 (R2) using a 5-point Likert scale, with 5 being the best; **A** R1—4 and R2—4, **B** R1—4 and R2—3, **C** R1—4 and R2—5, **D** R1—4 and R2—5

Based on the quadratic weighted Cohen’s kappa test, the interobserver agreement statistics indicate substantial to almost perfect agreement. In the 4DFB group, the values of ϰ for quality, respiratory artifacts, clarity, and sharpness average 0.76 in the arterial phase and 0.79 in the portal venous phase. In the BH group, these values average 0.77 in the arterial phase and 0.85 in the portal venous phase. Agreement tables for overall image quality are provided in Table 3. Table 4 compares the image quality of the 4D FreeBreathing sequence with that of the post-contrast-enhanced 3D T1w Vane mDixon sequence, and Fig. 2 presents the average data for the overall subjective image quality assessed by both readers. Here, the SNR and the subjective image quality were found to be equivalent. Figure 3 illustrates examples of image quality in different patients from the 4DFB group using 4D FreeBreathing, compared to the BH group undergoing cartesian dynamic 3D T1w mDixon imaging. Figure 4 demonstrates examples of image quality using 4D FreeBreathing during the portal venous phase, in comparison to 3D T1w Vane mDixon.

**Table 3:** Image quality scores for 4D FreeBreathing and cartesian dynamic 3D T1w mDixon by reader 1 (R1) and reader 2 (R2) using a 5-point Likert scale, with 5 being the best, including quadratic weighted Cohen’s kappa (ϰ)

	4D FreeBreathing						Cartesian dynamic 3D T1w mDixon					
Quality metrics	Arterial			Portal venous			Arterial			Portal venous		
	R1	R2	ϰ	R1	R2	ϰ	R1	R2	ϰ	R1	R2	ϰ
Diagnostic image quality	4.5 ± 0.7	4.4 ± 0.8	0.75	4.5 ± 0.6	4.5 ± 0.6	0.82	4.0 ± 0.8	3.7 ± 1.0	0.85	3.9 ± 0.9	3.5 ± 1.1	0.84
Respiratory motion robustness	4.5 ± 0.7	4.4 ± 0.6	0.74	4.4 ± 0.7	4.3 ± 0.7	0.65	3.9 ± 0.9	3.4 ± 1.1	0.75	3.8 ± 1.1	3.7 ± 1.2	0.90
Hepatic vessel clarity	4.7 ± 0.67	4.4 ± 1.1	0.72	4.5 ± 0.7	4.5 ± 0.8	0.85	4.1 ± 0.8	4.1 ± 1.1	0.74	3.8 ± 1.1	3.8 ± 1.3	0.74
Hepatic edge sharpness	4.7 ± 0.5	4.6 ± 0.6	0.81	4.5 ± 1.0	4.7 ± 0.5	0.82	4.1 ± 0.7	3.9 ± 1.2	0.75	3.9 ± 1.2	3.6 ± 1.2	0.90

**Table 4:** Image quality scores for 4D FreeBreathing and 3D T1w Vane mDixon by reader 1 (R1) and reader 2 (R2) using a 5-point Likert scale, with 5 being the best, including quadratic weighted Cohen’s kappa (ϰ) and signal-to-noise ratio (SNR)

	4D FreeBreathing			3D T1w Vane mDixon		
Quality metrics	R1	R2	ϰ	R1	R2	ϰ
Hepatic edge sharpness	4.5 ± 1.0	4.7 ± 0.5	0.82	4.6 ± 0.6	4.6 ± 0.6	0.86
Hepatic vessel clarity	4.5 ± 0.7	4.5 ± 0.8	0.85	4.4 ± 0.9	4.2 ± 0.9	0.72
Respiratory motion robustness	4.4 ± 0.7	4.3 ± 0.7	0.65	4.6 ± 0.6	4.6 ± 0.6	0.66
Diagnostic image quality	4.5 ± 0.6	4.5 ± 0.6	0.82	4.4 ± 0.7	4.5 ± 0.7	0.77
Motion artifacts	13	13	0.96	13	14	0.94
SNR	820 ± 260			750 ± 180		*p* = 0.35

### Analysis of anesthetic protocols

General anesthesia was performed on seven patients in group 4DFB and eighteen patients in group BH (*p* < 0.001). All other patients underwent imaging with a natural airway, divided into sixteen patients with sedation and two patients without any anesthesia for group 4DFB, as well as two patients with sedation and five patients without any anesthesia in group BH (*p* < 0.001). Imaging time using the 4D FreeBreathing protocol was significantly shorter for examinations performed under general anesthesia or sedation, while no significant difference was observed in cases without anesthesia, as shown in Table 2.

## Discussion

In this retrospective study, fifty consecutive infants and young children were examined with dynamic abdominal MRI, separated into two groups, either using the innovative 4D FreeBreathing sequence or not. There was no significant difference between both groups in age, gender and reason for investigation. We found no significant difference in the mean dynamic T1-weighted imaging times for all subjects using either the 4D FreeBreathing sequence or the traditional breath-hold exams, confirming that the 4D FreeBreathing sequence does not require any additional time. However, it was observed that the overall examination time decreased significantly in the 4DFB group.

Fortunately, our results indicate a significant improvement in image quality using the 4D FreeBreathing sequence, which demonstrated a significantly higher SNRart and SNRpv compared to conventional dynamic exams with breath-hold. Additionally, the subjective image quality in both the arterial and portal venous phases was rated favorably in all aspects for the group examined with the 4D FreeBreathing sequence, with substantial or almost perfect agreement across all evaluated criteria. Notably, the diagnostic image quality improved from 77.6 to 89.6%, while respiratory artifacts decreased from 22.4 to 10.4%. Recent studies showed that contrast-enhanced free-breathing radial sequence results in superior image quality compared to the conventional cartesian breath-hold examination in pediatric patients [18] or in patients with limited breath-hold capacity [20, 21]. However, there is so far less data on dynamic free-breathing radial stack-of-stars T1w sequences in infants and young children. Nevertheless, a recent study involving children who underwent magnetic resonance urography was able to corroborate our findings [22].

While previous studies have already demonstrated a good feasibility and image quality of post-enhanced stack-of-stars acquisition [23, 24], our study demonstrates that the dynamic 4D FreeBreathing sequence is not inferior to the contrast-enhanced 3D T1w Vane mDixon sequences. When comparing the dynamic 4D FreeBreathing and the contrast-enhanced 3D T1w Vane mDixon sequence, both the SNR and the subjective image quality were equal, and no additional information was revealed by the 3D T1w Vane mDixon sequence compared to the venous phase of the 4D FreeBreathing sequence. Therefore, the former could be omitted, given the excellent image quality and respiratory artifact robustness of the 4D FreeBreathing sequence. This allows for additional reduction in examination time.

The majority of the pediatric patients in this study underwent MRI under general anesthesia or sedation due to their inability to remain still during awake MRI exams, given their age or developmental stage. It has been demonstrated that anesthetic exposure during pediatric MRI is positively correlated with the duration of the MRI scan, and the administration of intravenous contrast agents can lead to increased scan times [25]. The utilization of robust, high-quality imaging sequences such as 4D FreeBreathing, rather than lengthier conventional sequences, aligns with efforts to minimize anesthesia exposure. Additionally, 4D FreeBreathing eliminates the need for breath-holding during dynamic T1-weighted imaging, significantly reducing the depth of anesthesia required and eliminating the necessity of invasive airway insertion. As a result, in our study, significantly fewer patients from group 4DFB underwent general anesthesia, opting instead for sedation using the 4D FreeBreathing sequences. These changes contribute to decreasing both short-term and long-term risks associated with anesthesia in the MRI setting [26, 27].

This study has several limitations. Firstly, the patient cohort in this study is relatively small, owing to the specialized examination protocol involving dynamic imaging and the young age of the patients. No significant differences were identified between the two groups concerning age, gender, or the reason for investigation. Nevertheless, this limitation underscores the importance of continually advancing examination techniques in order to provide substantial benefits to our young patients. Additionally, variations in sequence parameters between the traditional MRI and the study MRI (4D FreeBreathing) may have impacted imaging and anesthesia times, although no formal changes in sequence parameters were documented in the protocols. All patients were scanned on 1.5-T MRI systems with standardized indication-based protocols installed on all scanners. Apart from the introduction of the 4D FreeBreathing and the 3D T1w Vane mDixon sequence, there were no significant changes to these protocols during the study period, and an image review confirmed that all patients underwent imaging with the same protocol for both radial and traditional exams. However, approximately half of the children in the BH group were examined using the MRI 1.5-T Achieva Nova Dual, which may present a limitation of the study. Unfortunately, in the retrospective data analysis, it was not possible to determine whether sequences had to be repeated or to identify the causes of any interruptions or extensions of scan times. Therefore, it was not possible to further investigate why the overall examination time was shorter in the 4DFB group compared to the BH group.

During any abdominal MRI examinations of pediatric patients, it is particularly important to conduct a brief yet robust examination, with a strong emphasis on minimizing respiratory and motion artifacts. Our findings could be particularly beneficial for older pediatric patients who struggle to follow breath-hold commands but may not require sedation or general anesthesia.

## Conclusion

Dynamic radial stack-of-stars 4D FreeBreathing abdominal MRI in pediatric patients provides improved feasibility and enhanced image quality compared to dynamic cartesian T1-weighted imaging breath-hold techniques and maintains constant dynamic T1-weighted imaging time. Additionally, it demonstrates comparable image quality with post-contrast 3D T1w Vane mDixon. These advantages are particularly beneficial for infants and young children undergoing sedation or general anesthesia for imaging purposes, as the non-necessity of breath-holding presents opportunities for improved patient cooperation and diminishes the reliance on general anesthesia in pediatric patients.

## Abbreviations

4DFB: Patient group using 4D FreeBreathing sequence
BH: Patient group using conventional dynamic T1w sequence with breath-hold
DICOM: Digital Imaging and Communications in Medicine
DWI: Diffusion-weighted imaging
MRI: Magnetic resonance imaging
SNR: Signal-to-noise ratio
SNRart: Signal-to-noise ratio for arterial phase
SNRpv: Signal-to-noise ratio for portal venous phase
SPIR: Spectral presaturation with inversion recovery
